# Acute Descending Mediastinitis: An Unusual Presentation

**DOI:** 10.7759/cureus.27302

**Published:** 2022-07-26

**Authors:** Mohamed M Elagami, Moutaz Ghrewati, Ahmed Sharaan, Tarek Elzomor

**Affiliations:** 1 Internal Medicine, St. Joseph's University Medical Center, Paterson, USA; 2 Internal Medicine/Hospital Medicine, St. Joseph’s Wayne Hospital, Wayne, USA; 3 Critical Care Medicine, St. Joseph's University Medical Center, Paterson, USA; 4 Biological Sciences, New Jersey Institute of Technology, Newark, USA

**Keywords:** polymicrobial, retropharyngeal space, cervical fascia, mediastinum, descending mediastinitis

## Abstract

Descending necrotizing mediastinitis is believed to be a rare disease in an era where antibiotics have lowered the incidence of fulminant infections worldwide. Mediastinitis is the swelling and inflammation of the mediastinum, which is the central compartment of the thoracic cavity that contains the heart, thymus gland, parts of the esophagus, trachea, and other organs. Patients with acute descending mediastinitis can present with a wide spectrum of symptomatology including chills, high fever, tachycardia, dyspnea, nonproductive cough, retrosternal pain, hypotension, and Hamman sign. The deep neck infections found usually originate from infection at other primary sites, most often within the pharynx or oral cavity. It is commonly accepted that the cervical fascia is divided into three layers: the superficial, middle, and deep layers, and these layers divide the deep neck into multiple spaces. Invasion of the neck infections to surrounding tissues, including mediastinum, is limited due to enriched lymphatics that drain the area. Therefore, additional risk factors should interplay when the infections disseminate. Mediastinitis typically manifests as inflammation and swelling in the mediastinum, but our case was unique as the initial presentation was bilateral flank pain. Our goal is to raise awareness about this rare yet very serious complication.

## Introduction

Descending necrotizing mediastinitis is believed to be a rare disease in an era where antibiotics have lowered the incidence of fulminant infections worldwide. However, in developing countries, the incidence may be still relatively high. The delay in diagnosis and treatment has been associated with rapidly spreading infection, with a high mortality rate of up to 40% [1]. Patients with acute descending mediastinitis can present with a wide spectrum of symptomatology. The most common clinical manifestations include chills, high fever, tachycardia, dyspnea, nonproductive cough, retrosternal pain, hypotension, and Hamman sign. If a cervical abscess is present, the patient may complain of dysphagia, odynophagia, dysphonia, regurgitation, edema of cervical skin, and trismus. Our case is unique as the patient’s initial complaint was bilateral flank pain which represents an extremely rare presentation of acute descending mediastinitis. Our goal is to raise awareness about this rare yet very serious infectious disease and alert clinicians to the fact that it may present with atypical symptoms. Based on a literature review, we also discuss the diagnosis and treatment of this rare condition.

## Case presentation

Our patient is a 34-year-old female with no significant past medical history, who presented with a complaint of nonradiating bilateral flank pain that started one day prior to presentation. The pain was described as constant and throbbing in nature and was associated with nausea and a few episodes of clear emesis. The patient denied diarrhea, constipation, or urinary symptoms.

The patient also endorsed flu-like symptoms that began three days prior to presentation including generalized weakness, headache, subjective fever, dry cough, and sore throat. The patient, however, denied any recent sick contacts or travel. A review of symptoms was positive for posterior neck pain and left-side pleuritic chest pain that started three days prior to presentation. The patient denied prior exercise intolerance or any difficulty breathing.

On admission, vital signs were normal except for tachycardia of 110 beats per minute (BPM). Physical examination revealed an ill-appearing patient with erythematous tonsils, but no exudate was noted. A neck exam was significant for neck stiffness and right anterior cervical lymphadenopathy that was tender to touch. An abdominal exam revealed right-sided costovertebral angle (CVA) tenderness. Otherwise, the rest of the physical exam was normal. The blood work was significant for leukocytosis with bandemia (Table 1).

**Table 1:** Initial bloodwork results and reference ranges

Name of test	Reading	Reference range
White blood cells	21.4 K/mm^3^	4.5-11
Hemoglobin	11.3 g/dl	12-16
Hematocrit	35.9%	36-42
Platelets	242 K/mm^3^	140-440
Mean corpuscular volume	78.6 fL	80-100
Red cell distribution width	14.9%	0.5-16.5
Neutrophils	83%	36-75
Band cells	12%	0-10
Lymphocytes	3%	24-44
Monocytes	3%	4-10
Eosinophil	0%	0-5
Basophil	0%	0-2
Name of test	Reading	Reference range
Na+	135 Meq/L	135-145
K+	3.7 Meq/L	3.5-5
Chloride	99 Meq/L	98-107
Bicarbonate	24 Meq/L	21-31
Blood glucose	132 mg/dl	70-105
Blood urea nitrogen	12 mg/dl	7-23
Creatinine	0.91 mg/dl	0.60-1.30
Calcium	9.4 mg/dl	8.6-10.3
Total protein	7.2 g/dl	6.4-8.4
Albumin	3.9 g/dl	3.5-5.7
Alkaline phosphatase	116 IU/L	34-104

Initially, the patient underwent computed tomography (CT) scan of the abdomen that was significant for hepatomegaly only. However, it incidentally showed left pleural effusion in addition to diffuse fat stranding and fluid throughout the anterior mediastinum. The aforementioned findings mandated a CT scan of the chest that was notable for moderate bilateral pleural effusions along with redemonstration of extensive fat stranding and fluid in the anterior mediastinum. Additionally, it incidentally showed a left peritonsillar/pharyngeal abscess measuring 2.1 cm and several enlarged lymph nodes bilaterally in the neck. Further imaging, CT scan of the neck confirmed the presence of the left peritonsillar/pharyngeal abscess.

Based on the patient’s presentation, physical exam findings, leukocytosis with elevated bands on the blood work, and CT scans findings, we suspected a descending infection that originated from the peritonsillar abscess causing acute mediastinitis. Therefore, a septic workup was sent, and the patient was started on empirical antibiotics including piperacillin/tazobactam, clindamycin, and levofloxacin. Additionally, a multidisciplinary approach was necessary where an ear, nose, and throat (ENT) specialist and a cardiothoracic surgeon were consulted to drain the peritonsillar abscess and the mediastinal fluid, respectively.

Soon after admission, the patient's condition quickly deteriorated and she developed septic shock and respiratory failure requiring administration of vasopressors and endotracheal intubation. Later, the patient underwent mediastinoscopy along with bilateral chest tube insertion to drain the bilateral pleural effusion. Despite the septic workup not revealing any pathogen, the patient's condition responded to the empirical antibiotic regimen.

The hospital course was complicated by failure to extubate for which the patient underwent tracheostomy tube insertion and percutaneous endoscopic gastrostomy (PEG). Ultimately, the patient's condition improved and she underwent decannulation and removal of the PEG tube. She was then discharged from the hospital in good condition and was seen in the outpatient clinic with repeat CT imaging showing resolution of the mediastinal infection.

## Discussion

Mediastinitis is the inflammation of the thoracic cavity that contains the heart, thymus gland, parts of the esophagus, trachea, and other organs [2]. Descending necrotizing mediastinitis is believed to be a rare disease in an era where antibiotics have lowered the incidence of fulminant infections worldwide. However, in developing countries, the incidence may be still relatively high. The delay in diagnosis and treatment has been associated with rapidly spreading infection, with a high mortality rate of up to 40% [1]. Mediastinitis can present as acute or chronic. There may be many causes of acute mediastinitis such as microbial infections, esophageal rupture, radiation, inflammation of the lymph nodes, or cancer [3]. The microbial pathogens that cause mediastinitis vary based on the origin of the infection. For example, oral flora is found most commonly from infections originating from the oropharyngeal and esophageal sources [4]. Acute mediastinitis due to oropharyngeal infections typically starts with swelling, localized pain, and fever. As the infection makes its way to the mediastinum, symptoms such as chest pain, difficulty breathing, and odynophagia may arise [5]. Another cause of acute mediastinitis is infection secondary to cardiac surgery. The symptoms that develop after cardiac surgery are rather mild and nonspecific. Fever with sepsis may be the only finding. Any postcardiac symptoms usually arise two weeks after the operation and, most of the time, present with fever and chest pain [3]. Our case is unique as the patient’s initial complaint was bilateral flank pain which is an extremely rare presentation of acute descending mediastinitis. 

The deep neck infections found usually originate from infection at other primary sites, most often within the pharynx or oral cavity [6]. Consequently, pathogens may easily spread to the mediastinum through one of the three most clinically relevant spaces in the neck: the submandibular (and sublingual) space, the lateral pharyngeal (or parapharyngeal) space, and the retropharyngeal space [7].

It is commonly accepted that the cervical fascia is divided into three layers: the superficial, middle, and deep layers, and these layers divide the deep neck into multiple spaces. The superficial layer lies between the superficial cervical fascia and the muscles of the neck. It encircles the sternocleidomastoid and trapezius muscles as well as encloses the parotid gland. Inferiorly, this layer is continuous with the fascia of the pectoralis major anteriorly and with the thoracic portion of the trapezius and latissimus dorsi posteriorly. The middle layer is subdivided into the muscular and visceral portions. The muscular portion of the middle layers surrounds the strap muscles and the adventitia of the great vessels. The visceral portion of the middle layers surrounds the constrictor muscles of the pharynx and the esophagus. The middle layer superiorly attaches to the base of the skull and inferiorly extends to the pericardium. Similarly, the deep layer is also subdivided into two parts: the prevertebral and the alar. The prevertebral portions attach to the anterior part of the vertebral body and laterally to the transverse processes of the vertebrae. The alar portion of the deep layers is between the visceral and prevertebral parts of the middle layer. It surrounds the deep neck muscles as well as the cervical vertebrae and extends from the skull base into the mediastinum [5].

Nevertheless, invasion of the neck infections to the mediastinum is limited due to enriched lymphatics that drain the area. Therefore, additional risk factors should interplay when the infections disseminate. Kang et al. isolated factors that aid in spreading of the neck infections into mediastinum including being elderly, involvement of the retropharyngeal space, and comorbidities (hypertension, diabetes, liver cirrhosis, or chronic kidney disease) [7]. The patient in our presented case did not have any risk factors except for the involvement of the retropharyngeal space, which provided the route for the bacteria to disseminate [7].

A computed tomography (CT) scan is the most useful tool in diagnosing acute mediastinitis due to its ability to identify descending mediastinitis early and show varying degrees of tissue necrosis, soft tissue infiltration, localized collections, subcutaneous emphysema, and gas bands [7]. The rarity of acute descending mediastinitis mandates a low threshold of clinical suspicion. Estrera et al., in 1983, showed that there are four criteria to diagnose descending mediastinitis: 1) evidence of oropharyngeal infection, 2) radiographic characteristics of mediastinitis, 3) intraoperative or postmortem documentation of infection, and 4) establishment of a relationship between oropharyngeal process and mediastinitis [1].

In acute mediastinitis, the main treatment is surgery; in cases of acute mediastinitis dealing with the neck infections or esophageal perforations, either a head and neck surgeon or gastroenterologist should be seen. The first form of therapy after diagnosis of mediastinitis should be surgery as it is most effective [5]. Surgery may be needed in order to drain the infected fluid from the chest and/or repair an esophageal tear. Similarly, it is essential that a broad spectrum of antibiotics is quickly prescribed in order to attack multifarious possible pathogens. Medications to be taken for postsurgical acute mediastinitis should address nosocomial pathogens such as staphylococci, enterococci, and Gram-negative rods. For esophageal perforations, medications should cover oral pathogens such as staphylococci, streptococci, anaerobes, and enteric Gram-negative rods [5].

## Conclusions

In the era of multiple broad-spectrum antibiotics, the incidence of acute mediastinitis (descending necrotizing mediastinitis) had decreased tremendously. It is associated with a high mortality rate (42%) if recognized late or not treated correctly. Mediastinitis typically presents with chest pain associated with fever and dyspnea, but our case was unique in that the initial presentation was bilateral flank pain. Our goal is to raise awareness about this rare yet very serious infectious disease and alert clinicians to the fact that it may present with atypical symptoms. A low threshold, therefore, is required for quick diagnosis and intervention. The sooner the surgical therapy can be initiated, the higher the chance of survival. This in turn prevents serious complications such as pericardial effusion, sepsis, or even death. 

## References

[1] Estrera AS, Landay MJ, Grisham JM, Sinn DP, Platt MR (1983). Descending necrotizing mediastinitis. Surg Gynecol Obstet.

[2] (2021). Thorax. Encyclopædia Britannica. https://www.britannica.com/science/thorax.

[3] (2021). Medlineplus: Medical Encyclopedia: Mediastinitis. https://medlineplus.gov/ency/article/000081.htm.

[4] Cross MR, Greenwald MF, Dahhan A (2015). Esophageal perforation and acute bacterial mediastinitis: other causes of chest pain that can be easily missed. Medicine (Baltimore).

[5] Schooneveld Schooneveld, T. V. (2019, January 19 (2021). Infectious Disease Advisor: Mediastinitis. https://www.infectiousdiseaseadvisor.com/home/decision-support-in-medicine/infectious-diseases/mediastinitis/.

[6] Murray Murray, Alan; Meyers, Arlen Arlen (2021). Medscape: Deep neck infections. Medscape.

[7] Kang SK, Lee S, Oh HK (2021). Clinical features of deep neck infections and predisposing factors for mediastinal extension. Korean J Thorac Cardiovasc Surg.

